# The Wnt signaling pathway in tumorigenesis, pharmacological targets, and drug development for cancer therapy

**DOI:** 10.1186/s40364-021-00323-7

**Published:** 2021-09-06

**Authors:** Zhuo Wang, Tingting Zhao, Shihui Zhang, Junkai Wang, Yunyun Chen, Hongzhou Zhao, Yaxin Yang, Songlin Shi, Qiang Chen, Kuancan Liu

**Affiliations:** 1 Central Laboratory, Xiang’an Hospital of Xiamen University, Xiamen, Fujian 361102 P. R. China; 2 School of Medicine, Xiamen University, Xiamen, Fujian 361102 P. R. China; 3 Centre for Regenerative Medicine, Institute for Regeneration and Repair, The University of Edinburgh, Edinburgh, EH164UU UK; 4 Department of Biology, University of Rochester, Rochester, NY 14627 USA; 5 Cancer Centre, Faculty of Health Sciences, University of Macau, Macau, SAR China

**Keywords:** Wnt signaling, beta-catenin, Epigenetic modification, Tumor microenvironment, Drug development

## Abstract

Wnt signaling was initially recognized to be vital for tissue development and homeostasis maintenance. Further studies revealed that this pathway is also important for tumorigenesis and progression. Abnormal expression of signaling components through gene mutation or epigenetic regulation is closely associated with tumor progression and poor prognosis in several tissues. Additionally, Wnt signaling also influences the tumor microenvironment and immune response. Some strategies and drugs have been proposed to target this pathway, such as blocking receptors/ligands, targeting intracellular molecules, beta-catenin/TCF4 complex and its downstream target genes, or tumor microenvironment and immune response. Here we discuss the roles of these components in Wnt signaling pathway in tumorigenesis and cancer progression, the underlying mechanisms that is responsible for the activation of Wnt signaling, and a series of drugs targeting the Wnt pathway provide multiple therapeutic values. Although some of these drugs exhibit exciting anti-cancer effect, clinical trials and systematic evaluation should be strictly performed along with multiple-omics technology.

## Background

The Wnt signaling cascade is critical for tissue morphogenesis, homeostasis, and regeneration. Wnt signaling activation can go through the canonical or non-canonical pathway depending on the association with the transcription activator beta-catenin. Following the stimulation of the canonical pathway, beta-catenin is translocated from the cytoplasm to the nucleus [1]. By contrast, under the unstimulated condition, the levels of beta-catenin are relatively low in the cytosol due to the degradation complexes consisting of adenomatous polyposis coli (APC), glycogen synthase kinase-3β (GSK-3β), Axin, casein kinase 1(CK1). Degradation of beta-catenin is initiated by phosphorylation via GSK-3β and CK1, followed by β-transducin repeat-containing protein (β-TrCP) E3 ligase mediated ubiquitination [2], and the location and roles of components in the Wnt singalling pathway is summarized in Table 1. The non-canonical pathway includes several cascades, such as Wnt/planar cell polarity signaling, Wnt-cGMP/Ca^2+^ signaling and Wnt-RAP1 signaling [3]. Studies have shown that the non-canonical pathway plays a role in multiple physiological activities, including maintenance of stem cells in their niche [4].

**Table 1:** The location and function of components in Wnt signaling

Component	Subcellular location	Function
Wnt	Secreted to extracellular matrix	The ligands of the frizzled family, cause activation of TCF/LEF family transcription factors in the canonical Wnt signaling pathways
Dkk1	Extracellular region or secreted	Inhibiting the interaction between LRP5/6 and Wnt
Dkk3	Extracellular region or secreted	Antagonizes canonical Wnt signaling by inhibiting LRP5/6 interaction with Wnt, and promotes internalization of LRP5/6
LRP5/6	Plasma membrane, endoplasmic reticulum, membrane raft	Co-receptor as a member of the frizzled family of seven-transmembrane spanning receptors
Frizzled	Plasma membrane, endosome	Receptor for Wnt proteins which are coupled to the beta-catenin canonical signaling pathway
RNF43	Plasma membrane, endoplasmic reticulum, nucleus	A negative regulator of the Wnt signaling pathway by the ubiquitination, endocytosis, and degradation of Wnt receptor complex components Frizzled
ZNRF3	Plasma membrane	A negative regulator of the Wnt signaling pathway by the ubiquitination and degradation of Wnt receptor complex components Frizzled and LRP6
Dvl	Cytoplasm and cytosol, plasma membrane, cytoplasmic vesicle	Binding to the frizzled family members and transducing the Wnt signal
Axin1	Plasma membrane, cytoplasm and cytosol, nucleus, membrane	Component of the beta-catenin destruction complex that is required for down-regulating beta-catenin levels, modulates Wnt signal and controls dorsoventral patterning, enhances TGF-beta signaling and facilitates the phosphorylation of TP53
Axin2	Cytoplasm and cytosol	Inhibitor of the Wnt signaling pathway by down-regulating beta-catenin
APC	Plasma membrane, cytoskeleton, golgi apparatus, nucleus	Component of the beta-catenin destruction complex, serves as a negative regulator of Wnt signaling and promotes degradation of beta-catenin, plays a role in hepatocyte growth factor-induced cell migration and the localization of MACF1 to cell membrane
GSK3 β	Plasma membrane, cytoplasm and cytosol, nucleus	An active protein kinase and component of the beta-catenin destruction complex, phosphorylates the N-terminus of beta-catenin and promotes its degradation
Tankyrase	Cytoplasm and cytosol, cytoskeleton, golgi apparatus, nucleus, telomere	An activator of Wnt signaling pathway by poly-ADP-ribosylation of Axin1 and Axin2, regulates telomere length and vesicle trafficking
β-TrCP	Cytoplasm and cytosol, nucleus	Component of the beta-catenin destruction complex and mediates the ubiquitination of beta-catenin
beta-catenin	Plasma membrane, cytoplasm and cytosol, cytoskeleton, nucleus, adherens junction, cell junction, synapse	beta-catenin accumulates in the nucleus as a coactivator of TCF/LEF family of transcription factors based on the occurrence of Wnt ligands, leading to the activation of Wnt response genes, it is involved in the regulation of cell adhesion, chondrocyte differentiation and centrosome cohesion
Groucho	Nucleus	Transcriptional corepressor
TCF1	Nucleus	Transcriptional activator or repressor, beta-catenin binding, DNA binding
TCF3	Nucleus	DNA-binding transcription factor activity, repressing transcription factor binding, bHLH transcription factor binding
CBP	Cytoplasm and cytosol, nucleus	beta-catenin-TCF complex assembly, acetylates histones and non-histone proteins, specifically binds to phosphorylated CREB and enhances its transcriptional activity, serves as a coactivator or transcriptional coactivator
Porcupine	Endoplasmic reticulum	palmitoleoyltransferase activity by modifying Wnt protein with the attachment of palmitoleate, a step required for efficient binding to frizzled receptors

Wnt signaling also plays significant roles in tumorigenesis and progression. Dysregulated Wnt signaling activity promotes malignant transformation of stem/progenitor cell, leading to increased cell proliferation and abnormal differentiation. Wnt signaling also crosstalks with other signaling pathways (e.g. Hedgehog (Hh), Notch) to synergistically regulate tumor progression [5–7]. Abnormal Wnt signaling is associated with poor survival, stress responses, and drug resistance [8, 9]. In addition, canonical Wnt signaling modulates immune cell infiltration in tumors, rendering Wnt signaling a potential immunotherapy target [10]. Here, we review the role of Wnt signaling in cancer development in associated organs, we also review findings on targeting Wnt signaling for cancer therapy.

## Aberrant expression of Wnt signaling components during cancer development

Abnormal activation of Wnt signaling cascade is associated with tumorigenesis in several tissues including the esophagus and liver. Canonical Wnt signaling has been shown to promote the self-renewal of cancer stem cells (CSCs) [11, 12]. Esophageal squamous cell carcinoma (ESCC) and adenocarcinoma (EAC) are the two major cancer types of the esophagus. Dysregulated Wnt signaling was found in a significant number of cases of ESCC [13]. For example, WNT2 secreted by tumor fibroblasts promotes tumor cell proliferation and invasion via canonical signaling, and Wnt2-positive ESCC is correlated with lymph-node metastases [14]. In addition, beta-catenin expression was present in a heterogeneous pattern with prominent enrichment in the cell membrane of ESCC samples [15]. Moreover, the transcript and protein levels of beta-catenin and Wnt1 are elevated in ESCC carcinoma cells as compared to the neighboring normal tissues [16]. High levels of Wnt1 and beta-catenin predict lymph node metastasis, advanced pathological stage, and poor prognosis of patients. When combined with Bmi-1, Wnt1 and beta-catenin indicate a relatively worse prognosis [16]. Additionally, hypermethylation of Wnt antagonists/inhibitors including RUNX-3, DKK-3 (Dickkopf-3), and SFRP1 (Secreted Frizzled Related Protein 1) is associated with an elevated chance of ESCC recurrence. Thus, hypermethylation of the promoter of Wnt antagonists/inhibitors can potentially serve as a candidate indicator for ESCC treatment resistance [17].

Wnt signaling influences tumor growth at multiple levels. In squamous cell carcinoma (SCC) cell lines, beta-catenin is a key regulator for the expression of DeltaNp63 [18], a major isoform of p63 which is critical for basal cell proliferation and SCC development [19, 20]. Genes that contribute to regulation of Wnt family members are also associated with cancer progression. For example, Msi2 is a transcriptional regulator that regulates genes involved in development and cell cycle regulation [21, 22]. Overexpression of MSI2 enhances the activities of Wnt/beta-catenin and Hh signaling cascades, leading to increased proliferation of ESCC, epithelial-mesenchymal transition (EMT) and cell migration [23]. The expression levels of Msi2 are correlated with tumor size, differentiation, and lymph-node metastasis in patients with ESCC. Therefore, Msi2 is considered as an independent predictor for overall survival and disease-free survival [23]. In another study, organotypic 3D culture of primary human ESCC was used to show invasive cancer cells exhibiting activation of cyclin D1 and Wnt signaling [24]. Further study using dominant negative Mastermind-like1 to inhibit Notch signaling demonstrates Wnt signaling activation is Notch-independent [24].

In addition, inactivation of Wnt/beta-catenin and PI3K/AKT through depletion of Ras GTPase-activating protein SH3 domain-binding protein 1 (G3BP1) attenuates the proliferative, invasive and migration potential of esophageal carcinoma cells [25]. Wnt signaling can also be modulated by microRNA. For example, Cir-ITCH is a circRNA molecule that targets miR-214, miR-17, and miR-7. Cir-ITCH inhibits cell growth and proliferation of ESCC by enhancing the ITCH expression and inhibiting canonical Wnt signaling [26]. Interestingly, the transcript and protein levels of Dickkopf-1 (DKK1) that attenuate Wnt signaling activities, are up-regulated in esophageal cancerous tissues. Ectopic expression of DKK1 promotes cancer cell proliferation and invasion, although the underlying mechanisms remain to be determined [27]. Additionally, Naked cuticle homolog 2 (NKD2) is commonly methylated in gastric and breast carcinoma. Completely lost or reduced expression of NKD2 through methylation has also been observed in multiple ESCC cell lines and clinical samples. Further study showed that NKD2 attenuates the development of esophageal carcinoma through suppressing Wnt signaling in-vitro and in-vivo, suggesting that NKD2 methylation can potentially be used as a prognostic marker in esophageal carcinoma [28].

Abnormal Wnt signaling is also associated with tumorigenesis of EAC. For example, the mRNA levels of several members of the pathway are frequently altered during the progression of Barrett’s esophagus (BE) towards EAC. Changes in the methylation levels of the APC promoter were found in BE samples and 95% of EAC cases [29]. Methylation of SFRP1, which interacts with Wnts, followed by inhibition of the signaling activation was also detected in BE samples and EAC [29]. In EAC, beta-catenin nuclear-translocation was detected irrespective of APC expression; the upregulation of the WNT2 gene was found along with low-grade dysplasia to EAC [29].

## Multiple mechanisms underlie the stimulation of the Wnt/beta-catenin signaling cascade in cancer progression

### Activation of the Wnt/beta-catenin signaling cascade is driven by gene mutations

Mutations of CTNNB1 encoding beta-catenin are closely correlated with multiple cancers including hepatocarcinoma, pancreatic cancer, colorectal cancer (CRC), gastroesophageal junction carcinomas and gastric adenocarcinoma. The mutation of p.S45F in beta-catenin is a common mutation, leading to constitutively activated Wnt/beta-catenin signaling. This mutation has been found in a variety of solid tumors as a potential driver mutation, accounting for 3.3–10.4% of the total identified beta-catenin mutations [30]. Moreover, the somatic mutational profiling of 16 genes was analyzed in primary hepatocellular cancer. Somatic mutations occur in TP53 (33%) and CTNNB1 (22%) genes in 55% of the samples as identified through targeted deep sequencing. One protein, CTNNB1(H36P) encoded by the mutated CTNNB1 gene, enables resistance to the degradation of proteins and promotes cell proliferation. Of note is that mutations of TP53 and WNT/beta-catenin signaling cascades co-exist in hepatocellular carcinoma (HCC) [31]. In addition, CTNNB1 mutations are the only actionable genomic lesions in solid pseudopapillary neoplasms relative to other common subtypes of pancreatic tumors [32]. In animal models, mutations in CTNNB1 (exon 3) are predominant events among mutations of multiple genes in colon tumors induced by 2-amino-1-methyl-6-phenylimidazo [4,5-b] pyridine (PhIP) and dextrin sulfate sodium (DSS) in humanized CYP1A mice, one type of mice possessing human CYP1A gene to replace mouse Cyp1a gene. Whole exome sequencing revealed mutations on either codon 32 or 34 of exon 3 of the CTNNB1 gene in 39 out of 42 tumors. However, no mutations were detected in either APC or KRAS, suggesting that mutated CTNNB1 is the driver in colon cancer development induced by PhIP/DSS [33]. Besides cancers described above, driver mutations in CTNNB1 gene are also found in other types of cancer including lung cancer [34], endometrioid endometrial carcinoma [35], and mucosal melanomas [36] using whole-exome sequencing analysis and whole-genome sequencing analysis.

Mutation of APC is another important driver for tumor formation. Sixteen recurrently mutated genes were analyzed in 98 advanced CRC patients by next-generation sequencing. Multiple correspondence analysis (MCA) suggested that APC and TP53 mutations are close to the negative outlier group, whereas mutations in other members of WNT signaling are in proximity to the positive outliers. Moreover, patients with tumors harboring *TP53*mut/*APC*mut/*AMER1*wt/*TCF7L2*wt/*FBXW7*wt and *TP53*mut/*APC*mut/*AMER1*wt/*TCF7L2*wt/F*BXW7*wt/*SOX9*wt/*CTNNB1*wt genotypes had shorter progression-free survival. These two signatures are negatively correlated with the overall survival of CRC [37]. In another study, the combined effects of APC and BRAF mutation were tested in mice to determine the mutational landscape of WNT signaling regulators in BRAF mutant cancers. The results show that RNF43 gene is the most mutated WNT signaling regulator (41%), and mutations in the beta-catenin destruction complex are present in 48% of human CRC. 20.8% of CRC cases presented a truncation mutation in APC which is associated with early onset of tumor, advanced stage, and poor prognosis. Double mutations in APC and BRAF are associated with poorer prognosis than an individual mutation. These results suggest that the WNT signaling pathway is commonly mutated in CRC with BRAF mutation. Furthermore, WNT16 and MEN1 may drive aberrant WNT signaling in CRC [38].

Normal human intestinal epithelium-derived organoids have been used to study mutations of Wnt components and other genes associated with tumor. For example, organoids containing mutations of the five genes including SMAD4, APC, TP53, KRAS and/or PIK3CA, can grow independently of niche factors. These organoids also form tumors after implantation under the kidney capsule. Moreover, these organoids give rise to micrometastases post-injection into the mouse spleen, but fail to colonize in the liver [39]. Mouse models possessing numerous combined driver mutations including APC and other genes (KRAS, TRP53, TGFBR2 and FBXW7) have been generated for studying their contribution to the progression of intestinal tumors. Mutation of APC (∆716) causes intestinal adenomas and induces submucosal invasion when combined with TRP53 (R270H) mutation or a TGFBR2 deletion. Addition of the KRAS (G12D) mutation promotes EMT-like morphology and lymph vessel intravasation of the invasive tumors. By contrast, APC (∆716) combined with KRAS (G12D) and FBXW7 mutations induced EMT-like histology but failed to produce submucosal invasion. A combination of KRAS (G12D) with either APC (∆716) plus TRP53 (R270H) or TGFBR2 deletion caused highest incidence of liver metastasis with a genotype of APC (∆716) KRAS(G12D) TGFBR2 (−/−). These findings recapitulate the up-regulated genes observed in human metastatic CRC [40].

The Wnt antagonists SFRP1, SFRP2, DKK2 and Wnt inhibitory factor-1 (WIF-1) are hypermethylated during the transition from colorectal adenoma to carcinoma, whereas mutations in BRAF, APC, and KRAS occur at the transition from normal to adenoma phases, these events may drive the colorectal cancer formation [41]. Among 145 mutations within 31 genes detected in gastroesophageal junction carcinomas and gastric adenocarcinoma, mutations in APC and CTNNB1 are more prevalent among gastric carcinomas, with more than three driver mutations detected in gastric carcinomas. However, TP53 mutations are the most prevalent abnormalities that were detected, especially in gastroesophageal junction cancer [42]. Notably, mutations in either APC or CTNNB1 were also found in colon cancer, leading to increased activity of beta-catenin-Tcf signaling [43].

RNF43 is a negative regulator of Wnt signaling, and mutation of RNF43 is frequently found in CRC and endometrial cancers [38, 44], gastric cancer [45], neoplastic cysts of the pancreas including intraductal papillary mucinous neoplasms (IPMNs) and mucinous cystic neoplasms (MCNs) [46, 47]. Most mutation in RNF43 are truncation mutation with a higher mutation rate in the MSI subtype than in the MSS subtype [45]. Increased Wnt signaling upon the inactivated mutation of RNF43 is key for the transformation phenotype in pancreatic cancer cell lines. Consistently, tumors with an inactivating mutation of RNF43 are more sensitive to Wnt inhibitors, which therefore can be potentially developed as a therapy [48]. TP53 is often mutated in colitis-associated cancer (CAC), while SMAD4, KRAS, and APC have not been commonly mutated. RNF43 is also somatically mutated in 11% of CACs that have been linked with chronic inflammation and long-lasting inflammatory bowel disease (IBD). Many CACs with mutated APC are sporadic colorectal cancers. The expression level of c-Myc along with its target genes is elevated in RNF43-mutated CACs, suggesting that RNF43 is an important CAC driver. Somatic mutations of RNF43 result in genetic variation and are associated with chronic inflammatory process and progression of carcinoma in around 10% of CACs [49]. In another study, mutations of RNF43 were found in 0–17% samples, while other members of Wnt signaling in gastric cancer include CTNNB1 (3.3–9.1%) and APC (3.3–14.9%) [50]. RNF43 is also a tumor suppressor gene in mucinous tumors of the ovary, and RNF43 mutation was found in 21% of mucinous ovarian carcinomas [51]. By contrast, the mutation rates of APC and CTNNB1 are 45.8 to 90.6% and 5 to 7.2% in CRC based on TCGA database, respectively [52, 53].

**Fig. 1:**
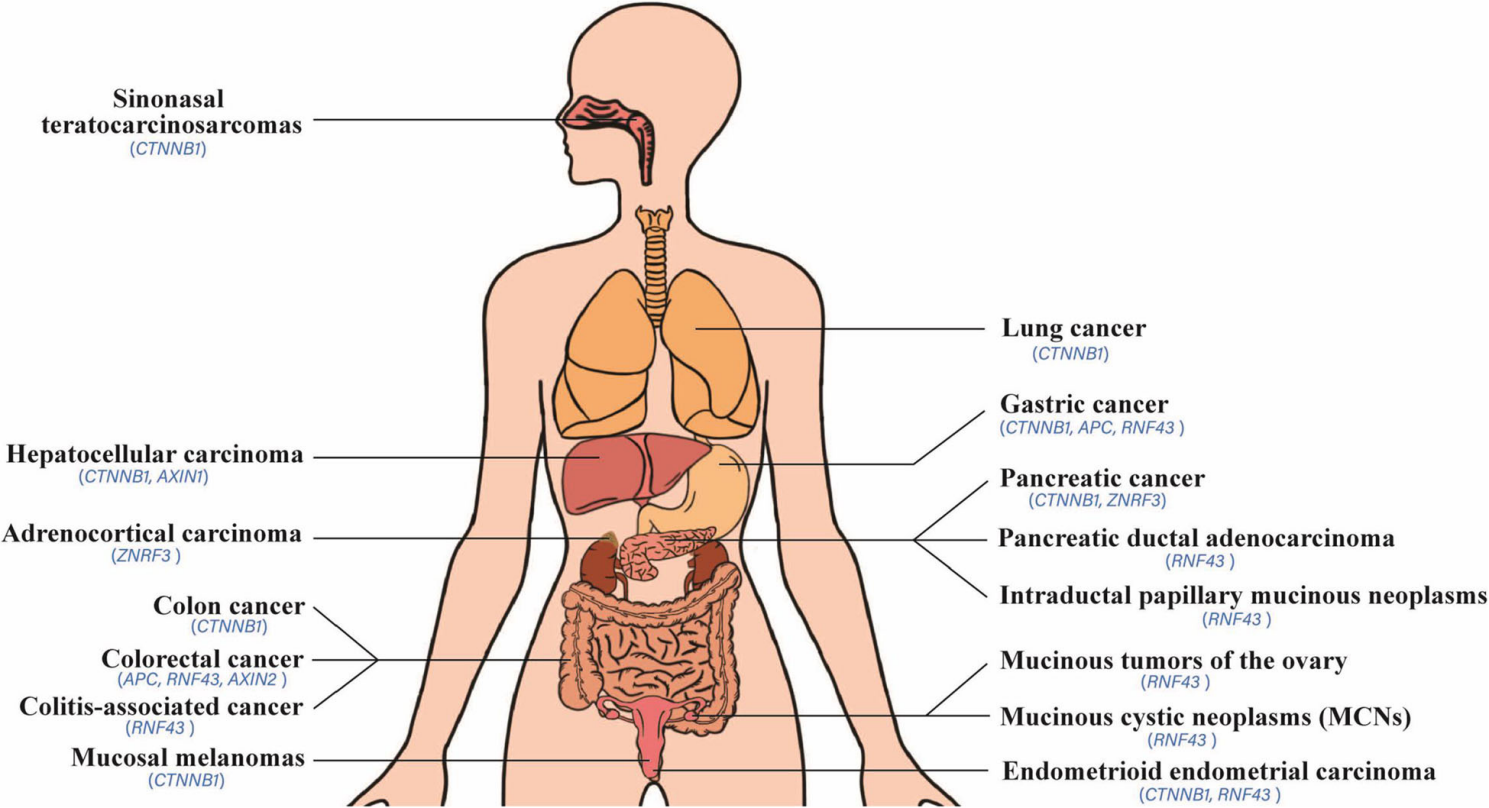
The distribution map of driver mutations on multiple human tissues within Wnt signaling

Mutations of other Wnt family members including ZNRF3 and AXIN. Mutation of ZNRF3, a Wnt signaling suppressor, is often found in adrenocortical carcinoma [54], while AXIN2 mutations induce a predisposition to colorectal cancer [55], its mutations are detected in colorectal cancer, finally leading to the accumulation of beta-catenin in the nuclei [56]. By contrast, AXIN1 mutation is found in HCC, overexpression of wild-type AXIN1 induces apoptosis in HCC and CRC cells with accumulated beta-catenin [57]. General driver mutations of the Wnt cascade in tumorigenesis are listed in Table 2 and Fig.1.

**Table 2:** Pivotal driver mutations of Wnt components occur in cancers

Mutated driver gene	Types of cancer	References
*CTNNB1*	sinonasal teratocarcinosarcomas	[30]
	hepatocellular carcinoma	[31]
	pancreatic cancer	[32]
	colon cancer	[33]
	lung cancer	[34]
	endometrioid endometrial carcinoma	[35]
	mucosal melanomas	[36]
	gastric cancer	[42]
*APC*	colorectal cancer	[37–41]
	gastric cancer	[42]
*RNF43*	pancreatic ductal adenocarcinoma	[43]
	gastric cancer	[45]
	intraductal papillary mucinous neoplasms (IPMNs), mucinous cystic neoplasms (MCNs)	[46]
	colorectal and endometrial cancers	[38, 44]
	IPMNs of pancreas	[47]
	colitis-associated cancer	[49]
	gastric cancer	[50]
	mucinous tumors of the ovary	[51]
*ZNRF3*	adrenocortical carcinoma	[54]
*AXIN2*	colorectal cancer	[55, 56]
*AXIN1*	hepatocellular carcinomas	[57]

### Wnt/beta-catenin signaling activation is caused by attenuating its inhibitor or via epigenetic modifications

Activation of the Wnt/beta-catenin cascade can also be obtained through suppressing its inhibitor. For example, expression of HOTAIR and secreted Wnt antagonist WIF-1 is inversely correlated in ESCC cell lines and tumor tissues. HOTAIR lowers the expression of WIF-1 by enhancing the methylation of H3K27 (one type of histone of WIF-1) at its promoter site, thereby triggering the activation of Wnt/beta-catenin signaling pathway [58]. Furthermore, the methylation levels of SOX17 (a Wnt signaling suppressor) increase along with the progression of esophageal cancer. SOX17 methylation is also associated with a history of alcohol consumption in esophageal cancer patients [59]. SOX17 methylation leads to the loss of SOX17 expression accompanied by increased expression of beta-catenin and redistribution. Re-expression of SOX17 via exposing 5-aza-2′-deoxycytidine attenuates TCF/beta-catenin-dependent transcription and proliferation of esophageal cancer cell lines. Conversely, SOX17 loss removes the normal inhibition of WNT signaling and enhances esophageal tumorigenesis. MicroRNA 141 can also downregulate the expression of SOX17 and stimulate the WNT signaling cascade [59].

Epigenetic regulation of the WIF-1 is also involved in gastro-intestinal tumorigenesis. Downregulation of WIF-1 caused by CpG island hypermethylation in the promoter region has been found in multiple cancer cell lines and esophageal, gastric, colorectal and pancreatic cancer tissues [60, 61]. Combined treatment of 5-aza-dC with the histone deacetylase inhibitor trichostatin A restores WIF-1 expression, and restoration of WIF-1 inhibits colony formation, cellular proliferation, and anchorage-independent growth of TE-1 ESCC cells or SW48 colon cancer cells [60]. In addition, elevated levels of WIF-1 promoter methylation occurred in EAC tissue samples compared to their matched normal epithelium. Hypermethylation of WIF-1 is more common in BE samples obtained from patients with EAC than in BE samples obtained from patients without EAC, suggesting that WIF-1 silencing caused by promotor hypermethylation underlies the progression of BE towards EAC. Consistently, restoration of WIF-1 in EAC cell lines suppresses the growth of the cells and sensitizes these cells to cisplatin [62].

Epigenetic inactivation of WIF-1 with promoter methylation also existing in ESCC cell lines and tissues. WIF-1 promoter methylation is commonly found in 46% ESCC tissues and 50% cell lines. Removal of promoter methylation with a demethylating agent such as 5-aza-2′-deoxycytidine results in the attenuation of cellular proliferation and migration, along with decreased activity of beta-catenin/T-cell factor-dependent transcription [63]. Consistently, ectopic expression of WIF-1 in nasopharyngeal carcinoma (NPC) and ESCC cells considerably attenuates the colony formation of tumor cells accompanied with significant down-regulation of beta-catenin protein. Therefore, epigenetic inactivation of WIF-1 contributes to the aberrant stimulation of the Wnt cascade in NPC and ESCC. Along this line, WIF-1 methylation may serve as a diagnostic tumor biomarker [64].

## Targeting Wnt signaling for therapeutic treatment of cancer

**Fig. 2:**
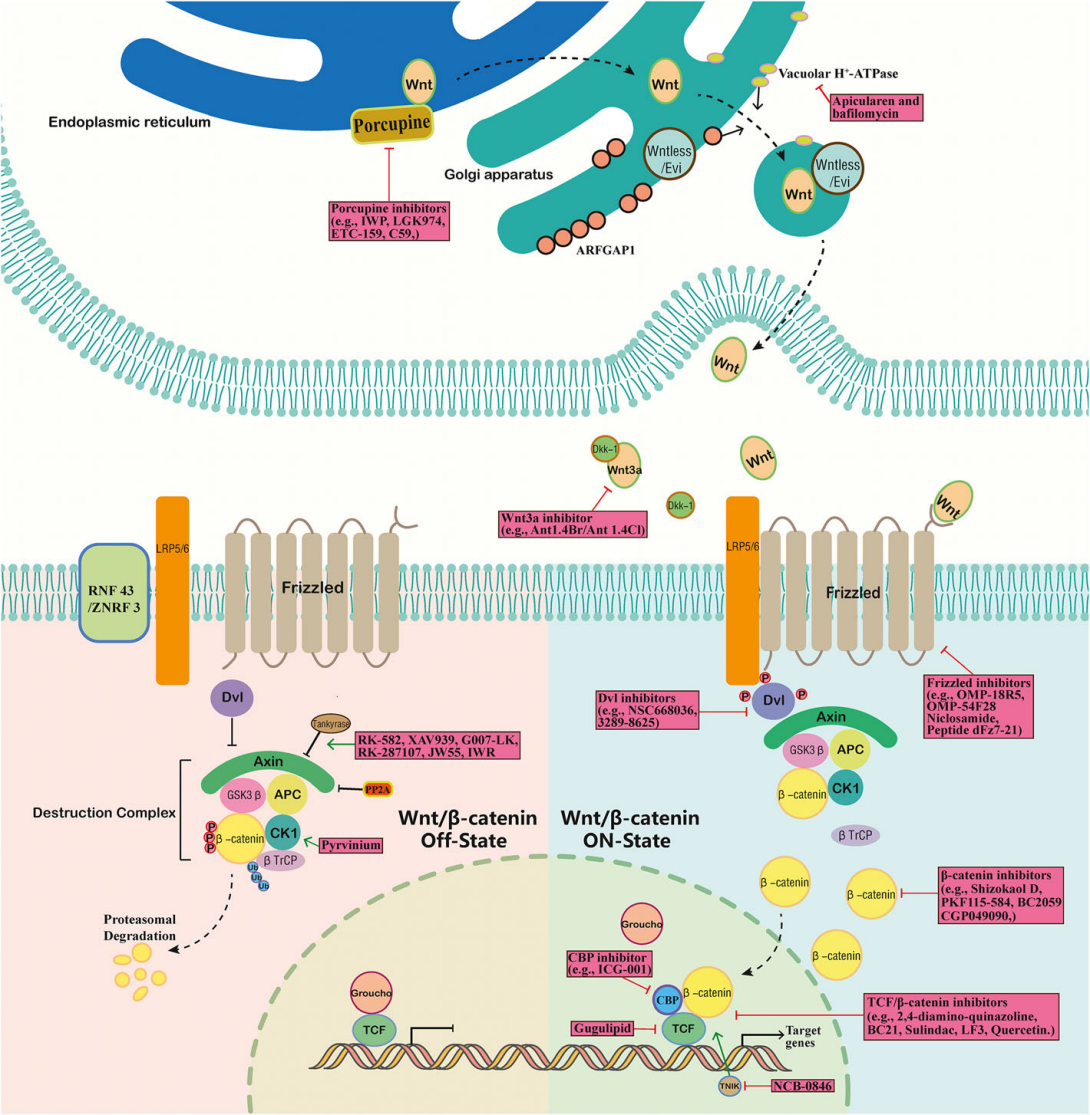
The Wnt signaling pathway and development of drugs against signaling components

Activation of Wnt signaling is correlated with tumorigenesis in multiple tissues. Therefore, a number of strategies targeting Wnt signaling as therapies have been proposed [8, 65], as summarized in Table 3 and Fig. 2. In Table 3, we described the representative the component of Wnt signal and the molecules are ongoing for drug development.

**Table 3:** Potential molecules and targets in drug development

Molecules	Targets	Inhibitor/activator of the target	Effect on signaling	Test in cancer	References
LGK974	Porcupine	inhibitor	inhibits	head and neck cancer, breast cancer, cervical cancer	[66]
IWP	Porcupine	inhibitor	inhibits	colorectal cancer	[67]
C59	Porcupine	inhibitor	inhibits	breast cancer	[68]
ETC-159	Porcupine	inhibitor	inhibits	colorectal cancer	[69]
OMP-18R5	Frizzled	inhibitor	inhibits	breast cancer, pancreatic cancer, colon cancer, lung cancer	[70]
OMP-54F28	Frizzled	inhibitor	inhibits	breast cancer, pancreatic cancer	[71]
Niclosamide	Frizzled 1	inhibitor	inhibits	osteosarcoma	[72]
Peptide dFz7–21	Frizzled 7	inhibitor	inhibits	not test	[73]
RK-582	Tankyrase, AXIN	activates AXIN	inhibits	colorectal cancer	[74]
XAV939	Tankyrase, AXIN	activates AXIN	inhibits	colorectal cancer	[75]
G007-LK	Tankyrase, AXIN	activates AXIN	inhibits	colorectal cancer	[76]
RK-287107	Tankyrase, AXIN	activates AXIN	inhibits	colorectal cancer	[77]
JW55	Tankyrase 1, tankyrase 2	activates AXIN	inhibits	colon cancer	[78]
IWR	Tankyrase, AXIN	activates AXIN	inhibits	colorectal cancer, cervical cancer	[79]
Shizokaol D	beta-catenin	inhibitor	inhibits	liver Cancer	[80]
NCB-0846	Traf2- and Nck-interacting kinase (TNIK)	inhibitor	inhibits	colorectal cancer	[81]
LF3	beta-catenin / TCF4	inhibitor	inhibits	colon cancer	[82]
2,4-diamino-quinazoline	beta-catenin / TCF4	inhibitor	inhibits	colorectal cancer	[83]
PKF115–584, CGP049090	beta-catenin	inhibitor	inhibits	colon cancer	[84]
BC21	TCF/beta-catenin	inhibitor	inhibits	colon cancer	[85]
Sulindac	TCF/beta-catenin	inhibitor	inhibits	familial adenomatous polyposis, colorectal cancer	[86]
NSC668036	PDZ domain of Dvl	inhibitor	inhibits	not test	[87]
3289–8625	PDZ domain of Dvl	inhibitor	inhibits	prostate cancer	[88]
BC2059	beta-catenin/ transducin β-like 1	inhibitor	inhibits	acute myeloid leukemia	[89]
Ant1.4Br/Ant 1.4Cl	Wnt3a	inhibitor	inhibits	not test	[90]
Pyrvinium	CK1α	activator	inhibits	colon cancer	[91]
Quercetin	beta-catenin / TCF4	inhibitor	inhibits	colon cancer	[85, 92]
ICG-001	cyclic AMP response element-binding protein (CBP)	inhibitor	inhibits	colon cancer	[93]
Apicularen and bafilomycin	Vacuolar H + -ATPase	inhibitor	inhibits	teratocarcinoma, neuroblastoma	[94]
Gugulipid	TCF-4	inhibitor	inhibits	breast cancer	[95]

### Blocking receptors/ligands of the Wnt signaling cascade

Blockage of Wnt signaling can be realized through ligands and receptors. While Frizzled (Fz)-related proteins (FRPs), Cerberus and WIF act by binding and sequestering Wnts, inhibitors against the receptors like LRP and Frizzled (FZD) also provide useful insights for cancer therapy [96]. For example, DKK1 inhibits Wnt signal transduction by binding to LRP, therefore blocking the interaction of the Wnt ligand to receptors [96–98]. LGK974 and IWPs are two potent and selective inhibitors of the Wnt palmitoyltransferase porcupine, resulting in reduced secretion of Wnt ligands. When applied, these two inhibitors decrease the phosphorylation of LRP6 receptors, and thereby inhibit Wnt/beta-catenin signaling [66, 99].

Another approach at attenuating Wnt signaling is through blockade of the receptor FZD [100], since Fzd 7 has been validated to be utilized as a candidate target to inhibit the growth of HCC cells with a small interfering peptides RHPDs [101]. Moreover, Soluble FZD 7 can interact with Wnt3 ligands, thereby competitively inhibiting the binding of a Wnt3 ligand to membrane FZD 7 receptors. This enhances the inhibitory effect on growth of tumor cells caused by doxorubicin via suppression of Wnt/beta-catenin signaling [102]. In addition, OMP-18R5 is a monoclonal antibody that directly binds to the extracellular segment of the FZD receptor members including FZD1, FZD2, FZD5, FZD7 and FZD8, it also reduces the phosphorylation of LRP6 and Wnt3A-induced accumulation of beta-catenin, more importantly, OMP-18R5 exhibits synergistic effect on tumor initiation when combining with chemotherapeutic agents after testing in xenograft model [70]. OMP-54F28, a fused protein that is generated by the fusion of a truncated FZD8 receptor with the IgG1 Fc region, has also been proved to block Wnt signaling and inhibit tumor growth and metastasis, and it also reduce the frequency of CSCs, more importantly, synergistic effect is achieved in breast cancer and pancreatic cancer after combination with other chemotherapeutic agents, such as gemcitabine [71].

Other strategies targeting Wnt ligands or receptors have also been developed. For example, RNAi compounds, monoclonal antibodies and small molecule inhibitors were proposed to target WNT2B [103] and the TNFα-WNT10B signaling loop [104]. Epigenetic disruption or overexpression of Wnt5a and epigenetic modification of its receptor ROR2 are also used to alter WNT5a and expression of ROR2, respectively [105, 106]. Therefore, a repertoire of inhibitors against Wnt receptors or ligands provide many potential choices for treatment of cancers with activated Wnt signaling.

### Targeting other intracellular molecules of the Wnt signaling pathway

Multiple intracellular components of Wnt signaling have been targeted for downregulation of the pathway. As a pivotal member of the Wnt signal pathway, reduction of intracellular beta-catenin or inhibition of the translocation of cytoplasmic beta-catenin to the nucleus, can effectively inhibit tumorigenesis and development, representing a pivotal therapeutic target for cancer treatment. Non-steroidal anti-inflammatory drugs (NSAIDs), such as aspirin and indomethacin, can down-regulate beta-catenin/TCF activity and their target genes cyclin D1 [107]. An important pathway for beta-catenin degradation has been explored involving Siah, SIP and Ebi, and the expression of Siah is induced by p53 protein, this pathway links genotoxic injury with the destruction of beta-catenin; thereby reducing activity of TCF/LEF transcription factors that contribute to cell cycle arrest [108]. Βeta-catenin and NFκB-p65 proteins bind to the promoter regions of MDR1 in imatinib-sensitive and resistant chronic myleoid leukemia (CML) cells. Nuclear beta-catenin, NFκB-p65 and Akirin-2 levels increase in cells with imatinib resistance. Therefore, targeting Akirin-2, NF-κB and beta-catenin genes may provide an opportunity to treat imatinib resistant CML [109].

Dishevelled (Dvl) is a key scaffold protein that transmits Wnt signals from the cell membrane to the cell. It consists of three conserved functional domains: DIX, PDZ and DEP. Among these domains, the PDZ domain directly binds to the FZD intracellular segment, thereby inhibiting GSK-3β activity, blocking beta-catenin phosphorylation, and causing the accumulation of beta-catenin in the nucleus. Finally, the Wnt signal is transmitted from the cell membrane into the cell [110]. The activity of Dvl is regulated by a series of proteins including and Dapper1 protein. Dapper1 promotes the degradation of Dvl, however, the binding of 14–3-3β to Dapper1 attenuates the ability of Dapper1, and the interaction between 14 and 3-3β and Dapper1 is dependent on protein kinase A (PKA)-mediated phosphorylation of Dapper1 at Ser-237 and Ser-827 [111].

AXIN is a negative regulator of Wnt signal and a scaffold protein involved in Wnt pathway signaling. It has multiple interaction sites with APC, GSK-3β, and CK1 to form beta-catenin destruction complexes, and it can also interact with other components of Wnt signals including Dvl and PP2A [112, 113]. Tankyrase promotes AXIN degradation through the ubiquitin-proteasome pathway and leads to the activation of Wnt pathway. Therefore, inhibition of tankyrase activity can prolong the decay of AXIN and promote the degradation of beta-catenin, leading to the inhibition of the Wnt/beta-catenin signaling pathway [74]. Several small-molecule inhibitor of tankyrase, such as RK-582 [74], XAV939 [75], G007-LK [76], RK-287107 [77], JW55 [78] and IWR [79], have been well developed and demonstrated to possess anti-tumor activity. When the colon cancer cells, SW480 and SW620, were treated with 5-fluorouracil (5-FU)/cisplatin (DDP) alone or in combination with XAV939, XAV939 achieved a synergistic effect on apoptosis with 5-FU/DDP in SW480 cells, suggesting XAV939 can significantly enhance apoptosis induced by 5-FU/DDP. This was accompanied by a changed level of beta-catenin protein, AXIN and CSC markers in colon cancer cells. Hence, AXIN may serve as a potential molecular target for reversing multidrug resistance in colon cancer [80].

Another study revealed that Delta isoform of the CK1 family of serine/threonine kinases (CK1δ), an important mediator of intracellular Wnt signaling, is always amplified, and overexpressed in a subset of breast cancer. Inhibition of CK1δ with knockdown or inhibitors leads to apoptosis, tumor regression and blocking nuclear accumulation of beta-catenin, thus it provides strategy for effective therapy for breast cancer expressing CK1δ [114]. when combined with LiCl or MG-132 treatment, SPINK5 overexpression impedes Wnt signaling by attenuating the phosphorylation of GSK-3β and promoting beta-catenin protein degradation. Consequently, SPINK5 overexpression attenuates the proliferation, migration and invasion of esophageal cancer cells [115].

In another study, significantly higher levels of beta-catenin and lower levels of miR-214 were detected in esophageal carcinoma specimens than paired adjacent tissue. The levels of beta-catenin and miR-214 were inversely correlated in esophageal cancer specimens. Further study revealed that miR-214 regulates the translation of beta-catenin. Overexpression and depletion of miR-214 inhibits and promotes cell growth and invasion in esophageal cancer cell lines, respectively [116]. Furthermore, the level of the sFRP-1 transcript is also regulated by Gli1 in gastric cancer cells. Hence, inhibition of Hh signaling results in the accumulation of Wnt1-mediated beta-catenin in the cytosol [5]. Additionally, other efforts have considered the functional restoration of WIF-1. This might also be developed as a new targeted therapy for the treatment of malignancy, and is correlated with Wnt signal activation.

### Targeting the beta-catenin/TCF4 complex and its downstream target genes in cancer

Beta-catenin regulates the expression of downstream target genes via interaction with TCF/LEF family members. Therefore, targeting the formation of beta-catenin-TCF/LEF complex or reduction in the levels of relevant proteins provides potential means to block the transcription of downstream target genes during cancer progression. Traf2- and Nck-interacting kinase (TNIK) is an important regulator of beta-catenin/TCF4 transcription complexes and is also required for the tumor-initiating capability of colorectal cancer stem cells. TNIK-deficient mice showed resistance to colon tumorigenesis induced by azoxymethane, and TNIK ^(−/−)^/APC ^(min/+)^ mutant mice form fewer intestinal tumors. The TNIK inhibitor NCB-0846 downregulates the levels of LRP5 and LRP6 and reduces the expression of the Wnt downstream targets AXIN2 and cMYC in HCT116 and DLD-1 colorectal cancer cells [81]. Therefore, small-molecule inhibitors against TNIK can be potential therapeutic reagents for treating colorectal cancer.

The 4-thioureido-benzenesulfonamide derivative LF3 also inhibits canonical Wnt signaling by directly interfering with beta-catenin/TCF-4 interactions. LF3 application reduced cell motility and self-renewal of colon CSCs; it also blocked tumor growth and induced differentiation in mouse xenografts [82]. In addition, Ethacrynic acid exerts anti-tumor effects by binding LEF-1, thereby breaking beta-catenin/LEF-1 transcription complexes and decreasing the expression of cyclin D1 and fibronectin in chronic lymphocytic leukemia (CLL) cells [117]. Significantly, since Ethacrynic acid is currently used as a diuretic in clinics, it can be readily repurposed to treat chronic lymphocytic leukemia and liver cancer [117, 118].

The Wnt downstream target S100A4 is involved in metastasis, promoting cancer cell migration and invasion. High levels of S100A4 predict metastasis and reduced survival in CRC patients. Therefore, S100A4 shRNA or small molecule inhibitors have been tested to treat CRC. In addition, a repurposed anti-helminthic drug niclosamide can inhibit the expression of S100A4 and a prospective phase II clinical trial was carried out for treating CRC patients [119, 120]. Several other reagents that target beta-catenin or its complex are also summarized in Table 3 and Fig. 2.

### Targeting Wnt signaling-mediated tumor microenvironment and immune response for cancer therapy

Oncogenic cascade activation in tumor cells affects the local anti-tumor immune reaction. Wnt signaling cross-talks with various immune cells, and thereby exhibits effects on tumor progression. It not only helps the maintenance and renewal of leucocytes, but also promotes immune tolerance, thereby limiting the anti-tumor response [121]. For example, Wnt/beta-catenin signaling activation reduces T-cell recruitment. In contrast, gain-of-function of MYC attenuates the activation and infiltration of T-cells [122]. Wnt/beta-catenin signaling activation leads to the exclusion of T-cell and resistance to immune therapy with anti-PD-L1/anti-CTLA-4 monoclonal antibody in autochthonous mouse melanoma models [123]. In addition, Tumor-infiltrating lymphocytes (TILs) and beta-catenin expression are both up-regulated in hormone receptor negative HER2-enriched and triple negative breast cancer (TNBC) subtypes. CD8(+) T-cells are the main effector cells in anti-tumor immunity. Interestingly, high levels of stromal TILs and CD8^+^ or FOXP3^+^ TIL subsets are linked with beta-catenin overexpression in breast carcinoma [124]. Correlations between the Wnt signaling activation and the absence of T-cell infiltration was recently explored in CRC. Studies revealed that tumors with high levels of beta-catenin exhibit decreased CD8+ T-cell infiltration. Therefore, a combination of PD-1-immunotherapy with beta-catenin targeting in CRC will likely be more helpful for therapeutic gains [125].

In addition to beta-catenin, other members such as WNT5a and DKK1 in the canonical Wnt signaling pathway were also explored for cancer immunotherapy. For example, nanoparticle-mediated trapping of WNT5a remodeled the immunosuppressive tumor microenvironment and enhanced the impact of immunotherapy, especially when combined with low-dose doxorubicin [126]. In addition, TGF-β1 silencing suppresses the DKK1 pro-invasive effect, and DKK1 enhances the invasion and migration of HCC via TGF-β1-mediated remodeling of the tumor microenvironment [127].

### Progress of drug development in clinical applications for targeting Wnt signaling

Although much progress in tumor suppress has been achieved upon targeting the member of Wnt signaling, no drug targeting this pathway has been approved in clinical applications due to potential side effects. However, some drugs which have been widely used for treating other diseases have been validated to suppress cancer progress, the strategy of analyzing their anti-cancer effect induced by current clinical drugs will save the time and cost. For example, niclosamide is an anthelminthic drug used for treating tapeworms in human, whereas accumulating studies also found that it can also target and inhibit the Wnt signaling in multiple types of cancer, such as ovarian cancer [128], colorectal cancer [129], prostate cancer and breast cancer [130], of note is that niclosamide also target other pathways including nuclear factor-kappaB (NF-kB) and Notch pathways. Besides niclosamide, other drugs comprising nigericin [131], psoralen [132], ethacrynic acid [118] and pyrvinium pamoate [133] are also involved in reducing the growth of cancer cells through inhibiting Wnt signaling. Clinically, nigericin is an antibiotic working by acting as an H^+^, K^+^, and Pb^2+^ ionophore, psoralen, ethacrynic acid and pyrvinium pamoate are used to treat vitiligo, hypertension and oedema, and helminth, respectively.

Another strategy of drug development against Wnt signaling is to screen novel inhibitors. So far, many potential drugs targeting Wnt signaling for cancer therapy have been studied in clinical trials at different stages, and representative drugs in specific phases are summarized in Table 4 after searching in https://clinicaltrials.gov/ct2/results. As shown in Table 4, the drug targeting a specific member of Wnt signaling can be used to treat multiple malignant cancer, and vice versa, patients with specific malignant cancer can also receive treatment programs with multiple drugs targeting different members of Wnt signaling.

**Table 4:** Representative drugs targeting Wnt signaling in clinical trials

Drug name	Target	Type of cancer	Phase	Therapeutic programe	NCT number
CWP232291	beta-catenin	acute myeloid leukemia, chronic myelomonocytic leukemia	phase 1	single group assignment	NCT01398462
Sinecatechins	beta-catenin	superficial basal cell carcinoma	phase 2/3	parallel assignment	NCT02029352
Foxy-5	Wnt-5a	metastatic breast cancer, colorectal cancer, prostate cancer	phase 1	single group assignment	NCT02020291
Foxy-5	Wnt-5a	metastatic breast cancer, metastatic colon cancer, metastatic prostate cancer	phase 1	single group assignment	NCT02655952
Rosmantuzumab (OMP-131R10)	Wnt/beta-catenin	metastatic colorectal cancer	phase 1	single group assignment. OMP-131R10 as a single agent and in combination with FOLFIRI	NCT02482441
PRI-724	Wnt/beta-catenin	acute myeloid leukemia, chronic myeloid leukemia	phase 1/2	parallel assignment	NCT01606579
PRI-724	Wnt/beta-catenin	pancreatic cancer	phase 1	all enrolled subjects will be treated with both PRI-724 and gemcitabine	NCT01764477
Onc201	Wnt/beta-catenin	advanced solid tumors, multiple myeloma	phase 1	single group assignment	NCT02609230
Mesalazine	Wnt/beta-catenin	sporadic colorectal adenoma	phase 2	parallel assignment	NCT01894685
Resveratrol	Wnt	colon cancer	phase 1	single group assignment	NCT00256334
Diclofenac	Wnt	basal cell carcinoma	phase 2	topical vitamin D3, diclofenac or a combination of both	NCT01358045
Genistein	Wnt	colon cancer, rectal cancer, colorectal cancer	phase 1/2	Genistein combined with FOLFOX or FOLFOX-Avastin	NCT01985763
Ipafricept (OMP-54F28)	Wnt	sarcomas, basal cell carcinoma, ovarian cancer, desmoid tumors and prostate cancer	phase 1	after OMP-54F28 is discontinued, all subjects will receive vitamin D3 and calcium carbonate twice daily	NCT01608867
WNT974 (LGK974)	RNF43	metastatic colorectal cancer	phase 1/2	phase l: dose escalation phase; phase ll: single group assessing the triple combination of WNT974, LGX818 and cetuximab	NCT02278133
ETC-1922159	Porcupine	advanced solid tumors	phase 1	ETC-1922159 as a single agent and in combination with pembrolizumab	NCT02521844
BI 905677	LRP5/6	different types of advanced cancer	phase 1	sequential assignment	NCT03604445
LY2090314	GSK-3β	advanced or metastatic cancer	phase 1	LY2090314 in combination with pemetrexed and carboplatin	NCT01287520
LY2090314	GSK-3β	leukemia	phase 2	single group assignment	NCT01214603
Vantictumab (OMP-18R5)	FZD	stage IV pancreatic cancer	phase 1	single group assignment, vantictumab in combination with nab-paclitaxel and gemcitabine	NCT02005315
Vantictumab (OMP-18R5)	FZD	non-small cell lung cancer	phase 1	single group assignment, vantictumab in combination with docetaxel	NCT01957007
Vantictumab (OMP-18R5)	FZD	metastatic breast cancer	phase 1	single group assignment, Vantictumab in combination with paclitaxel	NCT01973309
Niclosamide	Frizzled	prostate carcinoma	phase 1	niclosamide in combination with enzalutamide	NCT02532114
BHQ880	DKK-1	multiple myeloma	phase 2	parallel assignment, BHQ880 in combination with bortezomib and dexamethasone	NCT01337752
DKN-01	DKK-1	carcinoma of intrahepatic and extra-hepatic biliary system, carcinoma of gallbladder	phase 1	parallel assignment, DKN-01 in combination with gemcitabine and cisplatin	NCT02375880
Bortezomib	DKK-1	multiple myeloma	phase 4	prospective	NCT01026701
PRI-724	CBP/beta-catenin	pancreatic cancer	phase 1	single group assignment	NCT01764477
PRI-724	CBP/beta-catenin	myeloid malignancies	phase 1/2	parallel assignment, PRI-724 as a single agent and in combination with low dose ara-C therapy or dasatinib	NCT01606579

## Conclusion and perspectives

The Wnt signaling cascade not only controls the development and homeostasis of diverse organs, but also mediates the malignant transformation. Abnormal Wnt activity detected in tumor tissues is associated with tumor initiation, invasion, metastasis, and drug resistance. Further in vitro and in vivo modeling indicates that Wnt signaling can be abnormally activated through genetic and epigenetic modifications. Therefore, several strategies have been proposed for targeting this pathway, such as blocking receptors/ligands, targeting multiple molecules including intracellular molecules, beta-catenin/TCF4 complex and its downstream target genes, or tumor microenvironment and immune response mediated by Wnt signaling. Based on previous attempts, a series of potential drugs have been screened and developed, these drugs comprise small molecules, monoclonal antibody, and other molecules. In addition, some of these drugs also exhibit synergistic effect when combined with chemotherapy. Immunotherapy is a novel therapeutic method, and it also provides strong survival hopes for patients with cancer, especially for those with chemotherapy resistance, thus the combination of Wnt inhibitors with immunotherapy reagents are worth attempting.

Although a series of drugs against the components of Wnt signaling have been obtained, many more drugs targeting this pathway should also be explored with approved drug pool to save time and cost. Moreover, novel pharmacological molecules such as peptide can also be screened from current peptide pool. As described in our previous study, P42, a peptide targeting Sox2 protein, was successfully screened from a peptide library with BiFc method and immunoprecipitation, more importantly, this peptide has inhibitive roles on ESCC progression in vitro and in vivo [134]. Therefore, more novel molecules are worth trying to explore with multiple existing methods.

We have just begun to understand how mutations of the critical Wnt signaling genes (e.g. APC, beta-catenin) cooperate with other tumor-associated genes like p53 and PTEN etc. during initial tumor formation, growth, and metastasis. Similarly, findings of Wnt signaling activation through epigenetic modifications (e.g. methylation or acetylation) are also relatively new. We therefore need more comprehensive models to address the functional relevance of these new findings. In the future, new techniques including CRISPR/Cas9 gene editing, single cell analysis and high-resolution molecule imaging are key to facilitate the establishment of new models, allowing us for deep appreciation of the roles played by Wnt signaling in tumorigenesis. Accordingly, new therapeutic targets and therapies will likely emerge.

Currently, some clinical trials have also been performed with some drugs including niclosamide and LGK974 after searching at website: https://clinicaltrials.gov/ct2/home. However, since Wnt signal also acts in tissue development and homeostasis maintenance, it may also bring some side-effect when use these drugs, thus it is helpful for us to integrate these drugs with nanomaterials to avoid the side-effect on normal tissue development, thereby enhancing their exclusive anti-cancer effects on malignant tissues. Additionally, systematic evaluation on their anti-cancer effects should be strictly performed along with multiple-omics data integration, and the strict clinical studies should be accelerated.

## Data Availability

The materials supporting the conclusion of this review is included within the article.

## Abbreviations

APC: Adenomatous polyposis coli
BE: Barrett’s esophagus
CAC: Colitis-associated cancer
CBP: CREB-binding protein
CK1: Casein kinase 1
CK2: Casein kinase 2
CLL: Chronic lymphocytic leukemia
CML: Chronic myleoid leukemia
CRC: Colorectal cancer
CSCs: Cancer stem cells
Dkk: Dickkopf
DKK1: Dickkopf-1
DKK-3: Dickkopf-3
DSS: Dextrin sulfate sodium
Dvl: Dishevelled
EAC: Esophageal adenocarcinoma
EMT: Epithelial-mesenchymal transition
ESCC: Esophageal squamous cell carcinoma
FRPs: Frizzled (Fz)-related proteins
FZD: Frizzled
G3BP1: GTPase-activating protein SH3 domain–binding protein 1
GSK-3β: Glycogen synthase kinase-3
HCC: Hepatocellular carcinoma
Hh: Hedgehog
IBD: Inflammatory bowel disease
IPMNs: Intraductal papillary mucinous neoplasms
MCA: Multiple correspondence analysis
MCNs: Mucinous cystic neoplasms
MPNSTs: Malignant peripheral nerve sheath tumors
NKD2: Naked cuticle homolog 2
NSAIDs: Non-steroidal anti-inflammatory drugs
NPC: Nasopharyngeal carcinoma
PhIP: 2-amino-1-methyl-6-phenylimidazo [4,5-b] pyridine
RTKs: Receptor tyrosine kinase
SCC: Squamous cell carcinoma
SFRP1: Secreted frizzled related protein 1
TILs: Tumor-infiltrating lymphocytes
TNBC: Triple negative breast cancer
TNIK: Traf2- and Nck-interacting kinase
β-TrCP: β-transducin repeat-containing protein
WIF: Wnt inhibitory factor
WIF-1: Wnt inhibitory factor-1
